# Quantitative characterization of *Clostridioides difficile *population in the gut microbiome of patients with *C. difficile* infection and their association with clinical factors

**DOI:** 10.1038/s41598-020-74090-0

**Published:** 2020-10-19

**Authors:** Jieun Kim, Youna Cho, Mi-Ran Seo, Mi Hyun Bae, Bongyoung Kim, Mina Rho, Hyunjoo Pai

**Affiliations:** 1 Department of Internal Medicine, College of Medicine, Hanyang University, Seoul, 04763 Republic of Korea; 2 Department of Computer Science and Engineering, Hanyang University, Seoul, 04763 Republic of Korea; 3 Department of Laboratory Medicine, College of Medicine, Hanyang University, Seoul, 04763 Republic of Korea; 4 Department of Biomedical Informatics, Hanyang University, Seoul, 04763 Republic of Korea

**Keywords:** Computational biology and bioinformatics, Microbiology, Diseases

## Abstract

Objective was to analyse bacterial composition and abundance of *Clostridioides difficile* in gut microbiome of patients with *C. difficile* infection (CDI) in association with clinical characteristics. Whole metagenome sequencing of gut microbiome of 26 CDI patients was performed, and the relative abundance of *C. difficile* and its toxin genes was measured. Clinical characteristics of the patients were obtained through medical records. A strong correlation between the abundance of *C. difficile* and *tcdB* genes in CDI patients was found. The relative abundance of *C. difficile* in the gut microbiome ranged from undetectable to 2.8% (median 0.089)*.* Patients with fever exhibited low abundance of *C. difficile* in their gut, and patients with fewer *C. difficile* organisms required long-term anti-CDI treatment. Abundance of *Bifidobacterium* and *Bacteroides* negatively correlated with that of *C. difficile* at the genus level. CDI patients were clustered using the bacterial composition of the gut: one with high population of *Enterococcus* (cluster 1, n = 12) and another of *Bacteroides* or *Lactobacillus* (cluster 2, n = 14). Cluster1 showed significantly lower bacterial diversity and clinical cure at the end of treatment. Additionally, patients with CDI exhibited increased ARGs; notably, *bla*_*TEM*_*, bla*_*SHV*_ and *bla*_*CTX-M*_ were enriched. *C. difficile* existed in variable proportion of the gut microbiome in CDI patients. CDI patients with *Enterococcus*-rich microbiome in the gut had lower bacterial diversity and poorer clinical cure.

## Introduction

*Clostridioides difficile* infection (CDI) is one of the major hospital acquired infections^1^. Old age (> 65 years), use of antibiotics for non-CDI infections, and exposure to a hospital environment are some of the recognized risk factors for hospital acquired CDI^1,2^.

Antibiotics have a significant and long-lasting effect on the intestinal microbiota, and reduce colonization resistance against pathogens, including *C. difficile.* 16S rRNA gene sequence analysis of the gut microbiota of patients with CDI showed a highly variable bacterial composition of *Bacteroidetes* and *Firmicutes*, which is at odds with their predominance in the normal flora. Patients with recurrent CDI exhibited a lower species richness than patients with an initial episode of CDI and control subjects^3–6^. By altering the community structure of the gut microbiome, antibiotics alter the intestinal metabolome^3^; metabolic changes in bile acids and short-chain fatty acids are considered to play an important role in the development of CDI^3,6,7^.

Previous studies have indicated the importance of healthy gut microbiota and intact immune system in the pathogenicity of CDI^7–9^. Generally, CDI occurs in elderly patients with other comorbidities, and having variable states of immune function; these patients generally receive diverse combinations of antibiotics, which interfere with the gut microbiome^1,2,9^. The bacterial burden and toxin titres in stool do not show any correlation with clinical severity in mice and humans, but inflammatory markers do exhibit an association with clinical severity^9,10^. However, there are few studies analysing the relative abundance of *C. difficile* with respect to the bacterial diversity in the gut microbiome of patients with CDI^11^, and the influence of abundance of the organisms on the clinical presentation has not been investigated yet.

In this study, the population of *C. difficile* was measured using whole metagenome sequences of gut microbiome in patients with CDI in order to directly analyse the relationship with many clinical and microbiological variables. Firstly, metagenomes were used for analysing the changes in microbial composition in CDI patients. Secondly, we investigated the relationship of each genus or family in gut microbiome with *C. difficile* by comparing their relative abundance in each patient. Thirdly, despite the low number of CDI patients, the clinical variables such as severity or treatment results and relative abundance of *C. difficile* in gut microbiome were compared. Fourthly, the patients of CDI were clustered with respect to their metagenome profiles in the gut, and clinical and microbiological characteristics of these two clusters were evaluated. Finally, the distribution of antimicrobial resistance genes (ARGs) in gut of CDI patients was analysed in comparison with healthy individuals.

## Results

### Demographics and clinical characteristics

During the study period, a total of 26 CDI patients were enrolled. Table 1 presents the demographic and clinical characteristics of the patients. Median age of the patients was 66.5 years, and gender distribution was similar. The patients exhibited a mean score of 3 on the Charlson Comorbidity Index, and diabetes mellitus and malignancy were the common comorbidities. Of the 26 patients, 62% had a history of hospitalization within the previous 2 months and 92% had received antibiotics within 2 months before this episode. As for the clinical parameters at the time of diagnosis, 27% of the patients presented leukocytosis and 7.7% of the patients exhibited elevation of serum creatinine levels (1.5-times baseline). Severity index of CDI was assessed by the two aforementioned methods. Severe CDI was identified in 8 of the 26 patients (31%) based on leukocytosis and acute kidney injury^12^, and in 12 of the 26 patients (46%), the severity was identified based on the four factors described previously^13^. As for treatment of CDI, 24 out of the 26 patients were treated with metronidazole (20 [76.9%]) or vancomycin (4 [15.4%]), and recurrence of infection was observed in 7 patients (7/24 [29.1%]); the global cure rate was 54.2% (13/24). Though 3 patients (12.5%) died, none of the patients died because of CDI.

**Table 1:** Demographic data and clinical characteristics of patients of *Clostridioides difficile* infection.

Demographics and underlying diseases		
Age	Median (1Q, 3Q)	66.5 (59.8, 76.3)
Gender	Female—N (%)	13 (50)
Hospital day	Median (1Q, 3Q)	18.5 (7.8, 33.5)
**Charlson comorbidity index**	Median (1Q, 3Q)	3 (1, 5)
Chronic obstructive pulmonary diseases	N (%)	4 (15.4)
Asthma	N (%)	1 (3.8)
Malignancy	N (%)	7 (26.9)
Diabetes mellitus	N (%)	7 (26.9)
Chronic kidney disease	N (%)	6 (23.1)
Haemodialysis	N (%)	4 (15.4)
**History within 2 months—yes**		
Admission	N (%)	16 (61.5)
Use of antibiotics	N (%)	24 (92.3)
Use of proton pump inhibitor	N (%)	13 (50)
Use of probiotic	N (%)	5 (19.2)
**Clinical findings**		
WBC	Median (1Q, 3Q)	11,000 (7600, 16,350)
Albumin	Median (1Q, 3Q)	2.7 (2.3, 3.1)
Body temperature	Median (1Q, 3Q)	37.2 (37, 38)
Leukocytosis^1^	N (%)	7 (26.9)
Hypoalbuminemia^2^	N (%)	10 (38.5)
Fever^3^	N (%)	3 (11.5)
Acute kidney injury^4^	N (%)	2 (7.7)
**Severity score**		
**2 factors**^**5**^	Median (1Q, 3Q)	0 (0, 1)
Severe CDI by 2 factors	N (%)	8 (30.8)
**4 factors**^**6**^	Median (1Q, 3Q)	1 (1, 2)
Severe CDI by 4	N (%)	12 (46.2)
Toxin assay A&B	Positive	22 (84.6)
	Equivocal	3 (11.5)
	Negative	1 (3.8)
**Medication—initial**	N (%)	N = 26
Metronidazole		20 (76.9)
Vancomycin		4 (15.4)
None		2 (7.7)
Medication—final vancomycin	N (%)	7 (26.9)
Treatment duration	Median (1Q, 3Q)	13.5 (8.3, 15)
**Clinical response**	N (%)	N = 24
Cure at end of treatment		20 (83.3)
Mortality		3 (12.5)
Attributable mortality		0
Recurrence		7 (29.2)
Global cure		13 (54.2)

WBC, white blood cell.

^1^WBC count > 15,000 cells/mm^3^.

^2^Albumin level < 2.5 mg/dL.

^3^Temperature > 38.3 °C.

^4^Elevated serum creatinine: > 1.5 baseline.

^5^Sum of 2 factors ≥ 1: leukocytosis and acute kidney injury.

^6^Sum of 4 factors ≥ 2: age over 60, leukocytosis, fever, and hypoalbuminemia.

### Changes of microbial taxa correlated to CDI in gut microbiome

**Figure 1:**
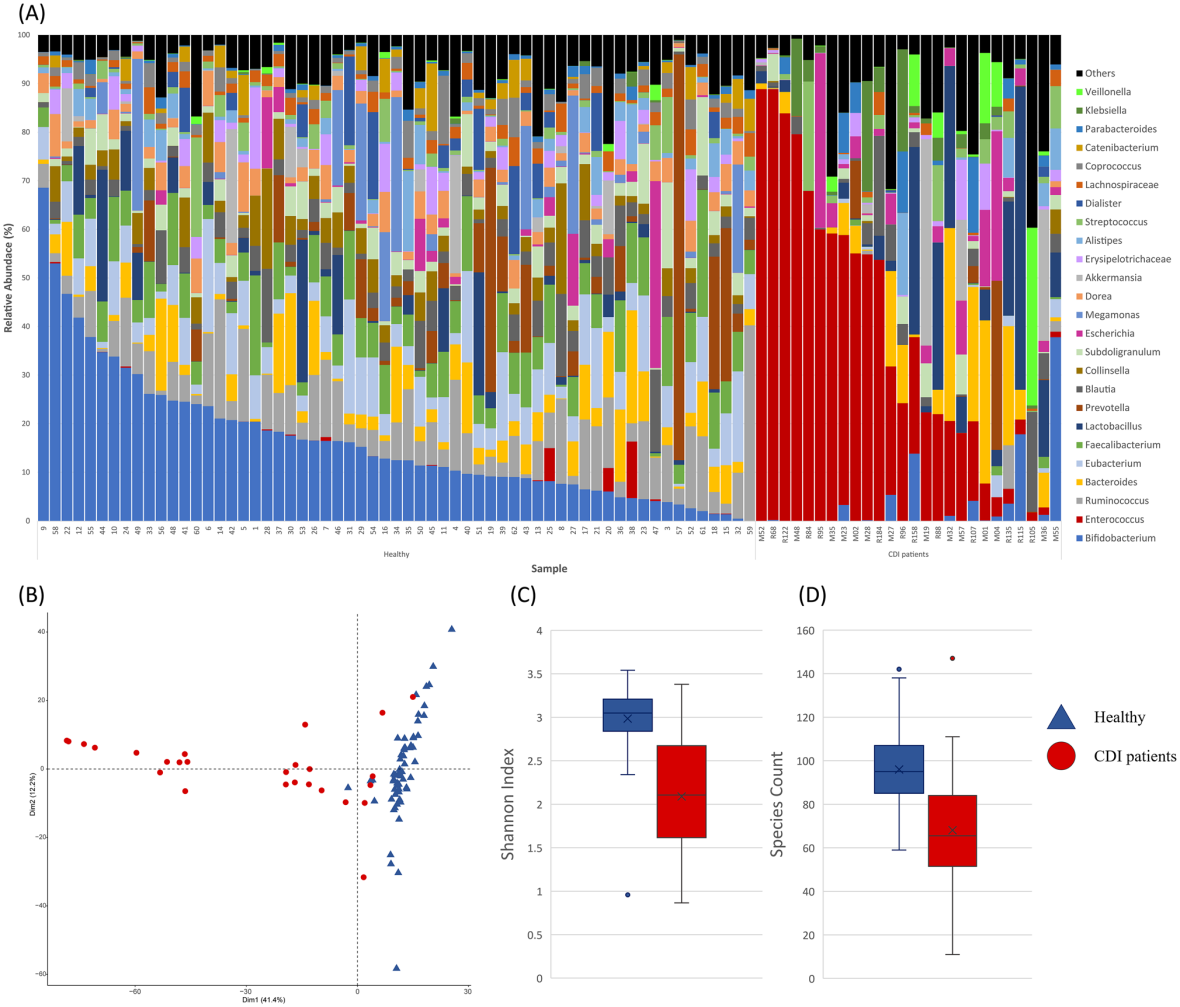
Bacterial composition of the gut microbiome in patients with *Clostridioides difficile* infection (CDI) and healthy individuals. (**A**) Bacterial composition at the genus level in the gut microbiome of patients with CDI and healthy people. (**B**) Principal component analysis of bacterial composition at the genus-level in patients with CDI and healthy individuals. (**C**) Shannon index of gut microbiome in patients with CDI and healthy people. (**D**) Species diversity of gut microbiome in patients with CDI and healthy people.

Figure 1A shows bacterial composition in the gut microbiome of 26 patients with CDI in comparison with that of 61 healthy individuals. At the genus level, 15 out of 25 detected genera (average proportion > 1%) showed significant decrease, and among them *Bifidobacterium, Ruminococcus, Eubacterium*, and *Faecalibacterium* showed a significant decrease in abundance in the gut of patients with CDI (*p* < 0.001 for all), whereas the abundance of *Enterococcus, Lactobacillus, Escherichia*, and *Klebsiella* increased (*p* < 0.001, *p* = 0.031*, p* = 0.002, and *p* < 0.001, respectively) (Supplementary Table S1). At the family level, 9 out of 16 detected families (average proportion > 1%) showed significant decrease; *Ruminococcaceae, Bifidobacteriaceae, Lachnospiraceae,* and *Eubacteriaceae* showed a decrease in abundance (*p* < 0.001 for all), whereas *Enterococcaceae, Lactobacillaceae*, and *Enterobacteriaceae* increased in abundance in the gut microbiome of patients with CDI (*p* < 0.001, *p* = 0.012 and *p* < 0.001, respectively) (Supplementary Table S2). Principle component analysis (PCA) on bacterial composition at genus level showed a clear separation between healthy individuals and patients with CDI (Fig. 1B), and the diversity of the gut microbiome was significantly lower in these patients (Fig. 1C,D).

### Relative abundance of toxigenic C. difficile in the gut microbiome of CDI patients

**Figure 2:**
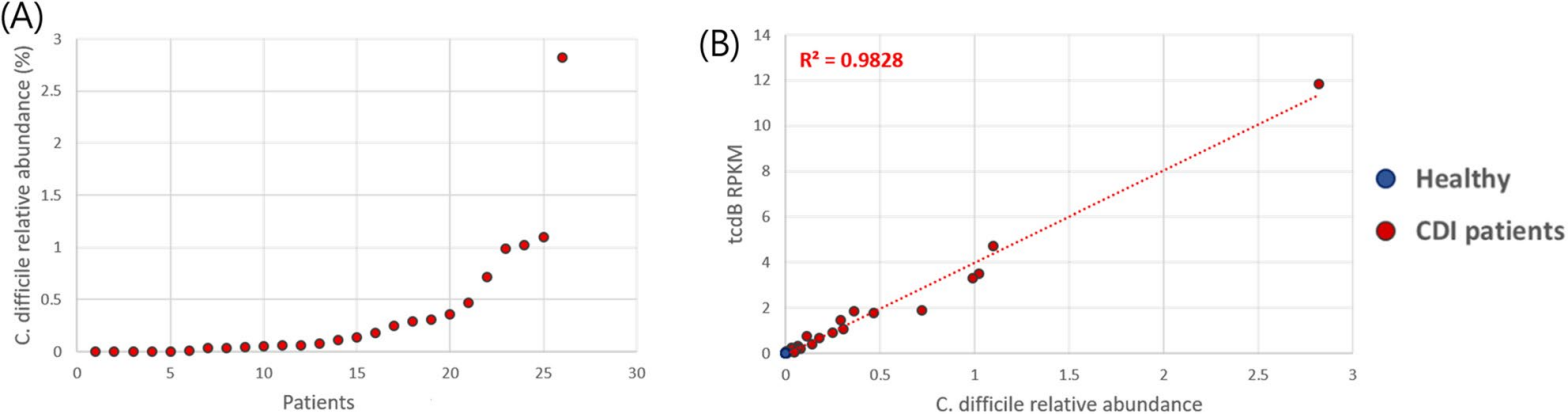
Relative abundance of *Clostridioides difficile* in the gut microbiome of 26 patients with CDI. (**A**) Distribution of *tcdB* abundance in the gut microbiota of 26 CDI patients; it ranges from 0 to 2.82%. (**B**) Correlation between *tcdB* abundance and abundance of *C. difficile* based on clade-specific marker genes.

In order to measure the relative abundance of toxigenic *C. difficile* in the gut microbiome, we measured the relative abundance of *tcdB* genes in the metagenome sequences of gut microbiome. The median relative abundance of toxigenic *C. difficile* in the gut microbiome was 0.089%, ranging from 0 to 2.82% (Fig. 2A). The relative abundance of *tcdB* genes measured by read mapping on *tcdB* and RPKM (read per kilobase million reads) showed a strong correlation with the abundance of *C. difficile* measured by MetaPhlAn clade-specific marker genes (r2 = 0.98) (Fig. 2B). For the reference, the relative abundance of *C. difficile* in non-CDI population is 0%.

### Factors associated with the relative abundance of toxigenic C. difficile in CDI patients

We observed that the relative abundance of toxigenic *C. difficile* in the gut microbiome shows an agreement with Ct value in real-time PCR of *tcdB* (rho =  − 0.605, *p* = 0.002) (Table 2). We analysed the clinical characteristics associated with the abundance of toxigenic *C. difficile* in the gut microbiome (Table 2)*.* Age, underlying diseases, and the use of antibiotics, proton pump inhibitor, or probiotics within 2 months from the episode of CDI were not associated with the abundance of toxigenic *C. difficile*. The abundance of toxigenic *C. difficile* had no effect on the occurrence of leukocytosis, hypoalbuminemia, or acute kidney injury. However, low numbers of toxigenic *C. difficile* in intestinal metagenomes were associated with fever (rho =  − 0.41, *p* = 0.038), and longer CDI therapy (rho =  − 0.405, *p* = 0.05). Treatment outcomes and the recurrence of CDI were not associated with the abundance of toxigenic *C. difficile* in the gut.

**Table 2:** Clinical findings associated with relative abundance of *C. difficile* in gut microbiome.

	Rho	*p* value
Demographics and underlying diseases		
Age	0.11	0.592
**Charlson comorbidity index**	0.155	0.45
Chronic obstructive pulmonary diseases	−0.078	0.704
Malignancy	0.318	0.113
Diabetes mellitus	−0.023	0.911
Chronic kidney disease	−0.104	0.615
**History within 2 months—yes**		
Admission	−0.026	0.898
Use of antibiotics	0	1
Use of proton pump inhibitor	−0.144	0.484
Use of probiotics	0.137	0.505
**Clinical findings**		
Leukocytosis^1^	0.012	0.955
Hypoalbuminemia^2^	0.084	0.682
Fever^3^	−0.41	0.038
Acute kidney injury^4^	−0.173	0.397
**Severity score**		
Severe CDI by 2 factors^5^	−0.006	0.978
Severe CDI by 4 factors^6^	−0.031	0.881
**Toxin test**		
Ct value of real time PCR for *tcdB*	−0.605	0.002
Toxin assay A&B	0.18	0.379
Treatment duration for CDI	−0.405	0.05
**Clinical response**		
Mortality	0.246	0.247
Recurrence	−0.093	0.666
Global cure	0.073	0.76

*p* value by Spearman’s rho correlation analysis.

^1^WBC count > 15,000 cells/mm^3^.

^2^Albumin level < 2.5 mg/dL.

^3^Temperature > 38.3 °C.

^4^Elevated serum creatinine: > 1.5 baseline.

^5^Sum of 2 factors ≥ 1: leukocytosis and acute kidney injury.

^6^Sum of 4 factors ≥ 2: age over 60, leukocytosis, fever, and hypoalbuminemia.

We observed the relationship between the abundance of microbial families or genera and the abundance of toxigenic *C. difficile* in the gut microbiome (Supplementary Table S3). At the genus level, the abundance of *Bifidobacterium* and *Bacteroides* showed a negative correlation with the abundance of toxigenic *C. difficile* (rho =  − 0.417, *p* = 0.034; rho =  − 0.403, *p* = 0.041, respectively). As these genera are the main constituents of the families, *Bifidobacteriaceae* and *Bacteroidaceae,* their populations also showed a negative correlation with the abundance of toxigenic *C. difficile* (rho =  − 0.411, *p* = 0.037; rho =  − 0.403, *p* = 0.041, respectively).

### Antibiotic resistance genes in gut microbiome of CDI patients

**Figure 3:**
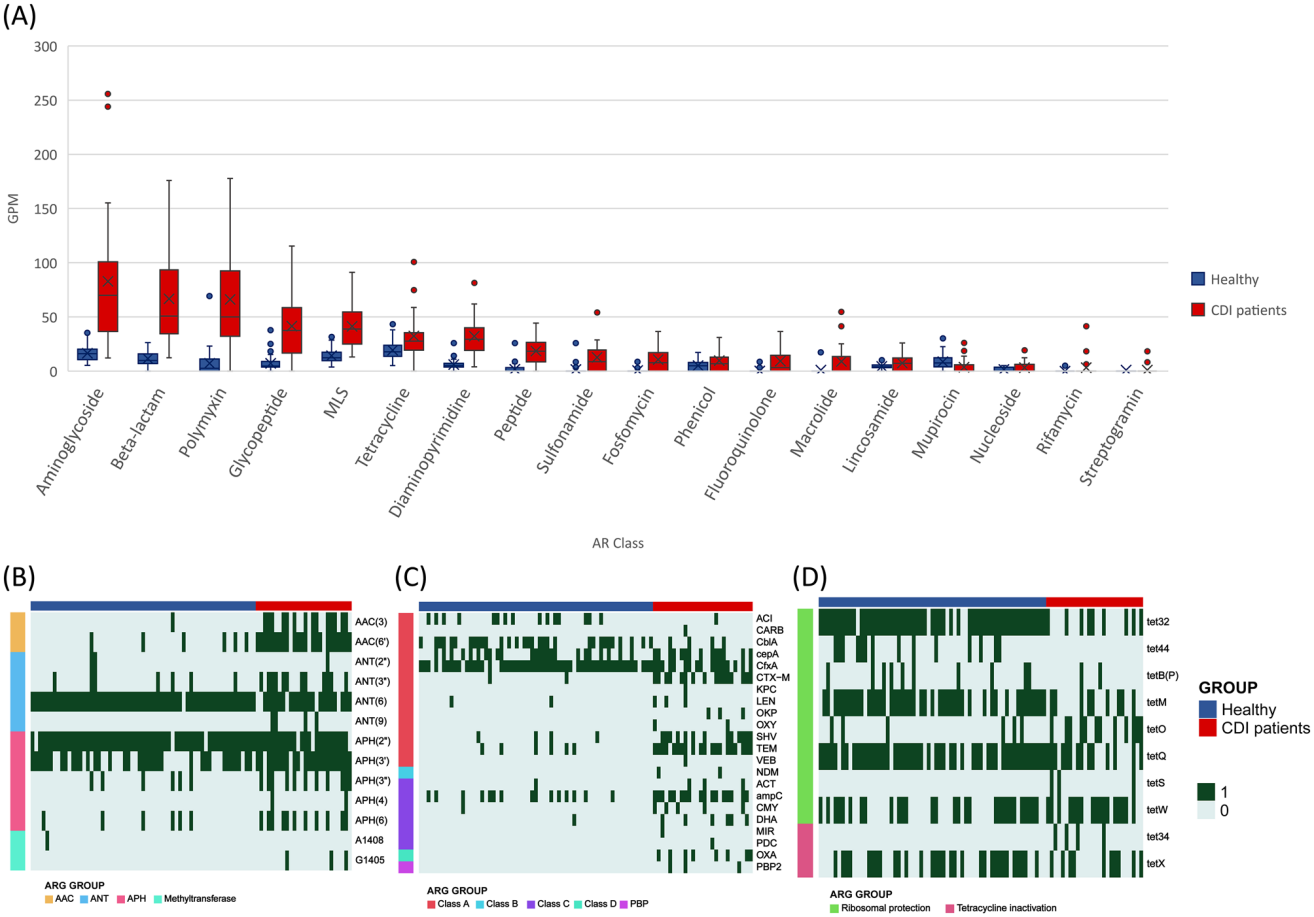
Differential distribution of antibiotic resistance genes in patients with *Clostridioides difficile* infection and healthy people. (**A**) Distribution of AR class in healthy individuals and patients with CDI (*p* value < 0.01 in t-test). (**B**) Differential distribution of beta-lactam resistance genes in patients with CDI and healthy people. (**C**) Differential distribution of aminoglycoside resistance genes in patients with CDI and healthy people. (**D**) Differential distribution of tetracycline resistance genes in patients with CDI and healthy individuals.

A total of 53 ARG families from 20 classes were screened in the gut microbiome. Compared to the microbiome of healthy individuals, the gut microbiome of the patients with CDI exhibited a fourfold higher abundance of ARGs. The resistome in the healthy population ranged from 49.7 to 292.5 GPM with a median value of 89.7, while in CDI patients, it ranged from 141.5 to 1095.8 with a median value of 356.7 GPM. Figure 3A presents the abundance of 15 ARG classes, which were significantly different between the healthy individuals and the CDI patients (*p* value < 0.01 in t-test). Notably, resistance genes against β-lactam, aminoglycoside, polymyxin, LMS, and glycopeptide were markedly enhanced in patients with CDI (5.1, 4.3, 18.5, 3.1, and 7.7-fold, respectively). Figure 3B–D present the differential prevalence of β-lactam, aminoglycoside, and tetracycline resistance genes in healthy individuals and patients with CDI patients. In particular, class A β-lactamase genes such as *bla*_*TEM*_, *bla*_*SHV*_, and *bla*_*CTX-M*_ genes were markedly enhanced; class C plasmid-mediated AmpC genes such as *bla*_*CMY*_ and *bla*_*DHA*_ were also enhanced in patients with CDI. Notably, KPC and NDM carbapenemase genes were observed in one and two patients, respectively. As for aminoglycoside genes, ANT(3′), AAC(3), and AAC(6′)-APH(2″) were markedly enhanced in the gut microbiome of CDI patients (Fig. 3C). However, tetracycline resistance genes showed only a slight increase (1.5-fold), and the distribution of individual tetracycline resistance genes was significantly different; *tet32, tet44, tetM*, and *tetQ* were fewer, but *tetO, tetS*, and *tet34* appeared more in the gut microbiota of patients with CDI than in the gut microbiota of healthy individuals (Fig. 3D).

### Distinct bacterial community in two different groups of CDI patients

**Figure 4:**
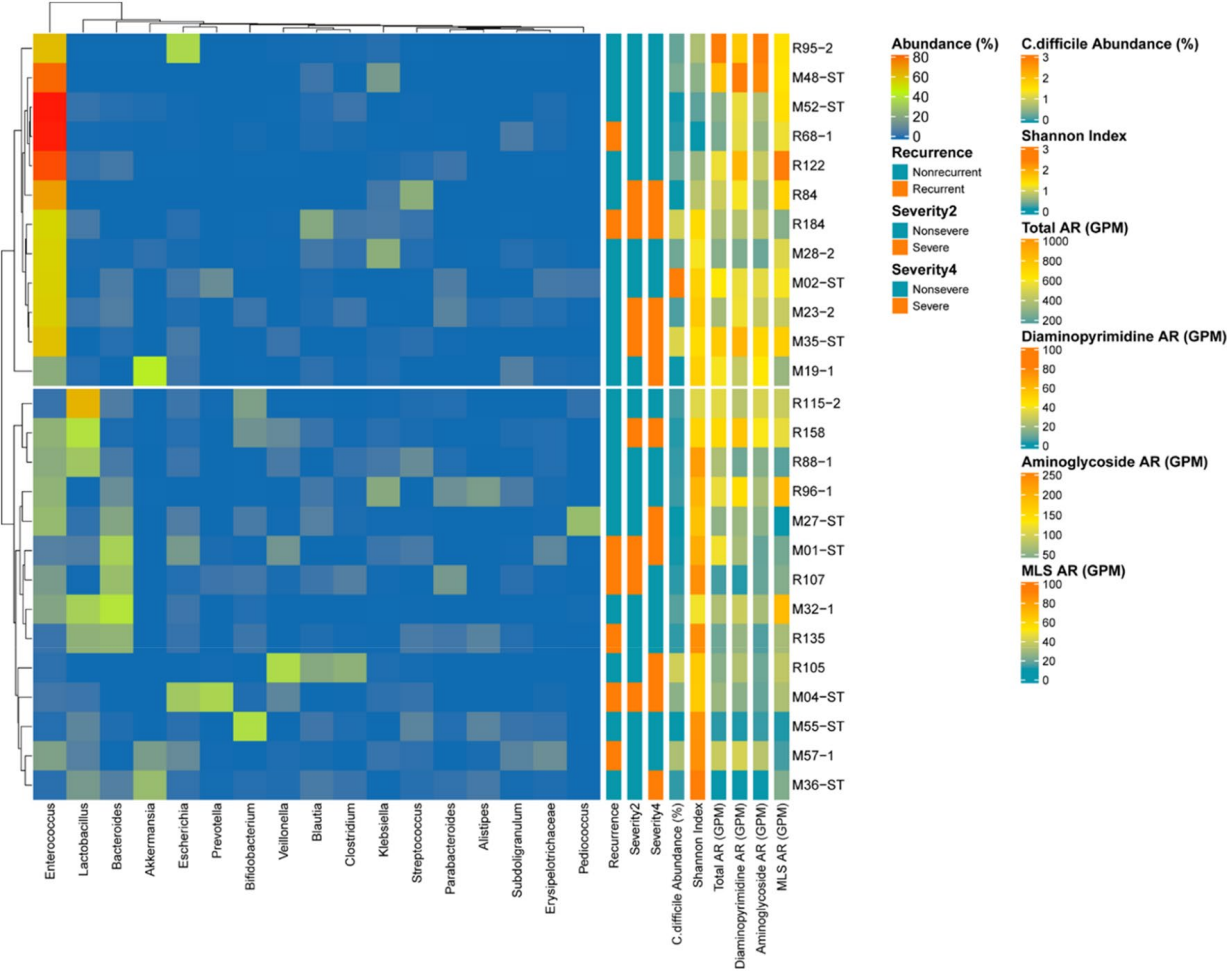
Clusters of patients with *Clostridioides difficile* infection with respect to the bacterial composition.

When the patients with CDI were clustered with respect to the bacterial composition, two different groups were observed with different major constituents in their bacterial communities (Fig. 4): a cluster of samples with high abundance of *Enterococcus* (cluster 1, n = 12), and a cluster with high abundance of *Bacteroides* or *Lactobacillus* (cluster 2, n = 14). The bacterial diversity was significantly low in cluster 1, compared to cluster 2 (*p* < 0.001), which was evident in the genus distribution shown in Fig. 4. The proportion of *C. difficile* was not different between the 2 clusters (*p* = 0.129). Interestingly, the abundance of ARGs was differentially distributed between the two groups. In particular, aminoglycoside, diaminopyrimidine, and LMS resistance genes were overrepresented in the patients with high abundance of *Enterococcus* (*p* value < 0.05). However, the recurrence status, severity scores, and the total abundance of ARGs did not show any significant difference between these two groups. Clinical characteristics were compared between the 2 clusters (Table 3); more patients in *Bacteroides* group took proton pump inhibitor (*p* = 0.018) or fluoroquinolone marginally (*p* = 0.065) within previous 2 months. Disease severity was not different between the 2 groups but clinical cure was achieved in more patients of *Bacteroides* group (*p* = 0.031) and all fatal cases came from the *Enterococcus* group.

**Table 3:** Comparison of clinical characteristics between the cluster with high abundance of *Enterococcus* (cluster 1, n = 12) and the cluster with high abundance of *Bacteroides* or *Lactobacillus* (cluster 2, n = 14) among *C. difficile* infections.

Clinical characteristics		*Enterococcus*	*Bacteroides*	*p* value
		N = 12	N = 14	
Age	Median (1Q, 3Q)	67 (59.3, 76.8)	66.5 (60.5, 76.8)	1*
Female	N (%)	4 (33.3)	9 (64.3)	0.116
**Charson comorbidity index**	Median (1Q, 3Q)	1.5 (1, 4.5)	3 (2, 5.3)	0.13*
Malignancy	N (%)	1 (8.3)	6 (42.9)	0.081
Diabetes mellitus	N (%)	3 (25)	4 (28.6)	1
Chronic kidney disease	N (%)	2 (16.7)	4 (28.6)	0.652
Leukocytosis^1^	N (%)	4 (33.3)	3 (21.4)	0.665
Hypoalbuminemia^2^	N (%)	6 (50)	4 (28.6)	0.422
Fever^3^	N (%)	0	3 (21.4)	0.225
Acute kidney injury^4^	N (%)	1 (8.3)	1 (7.1)	1
Hemodialysis	N (%)	0	4 (28.6)	0.1
**Severe CDI**	N (%)			
By 2 factors^5^		4 (33.3)	4 (28.6)	1
By 4 factors^6^		5 (41.7)	7 (50)	0.713
Treatment duration	Median (1Q, 3Q)	10 (7, 15)	14 (10, 16.5)	0.268*
Toxin assay A&B-positive	N (%)	10 (83.3)	12 (85.7)	0.579*
Relative abundance of *C. difficile* (RPKM)	Median (1Q, 3Q)	0.25549 (0.025646, 0.79769)	0.05952 (0.00629, 0.26057)	0.129*
**Medical history**				
Admission history within 2 months	N (%)	7 (58.3)	9 (64.3)	1
Proton pump inhibitor intake	N (%)	3 (25)	10 (71.4)	0.018
Probiotics intake	N (%)	3 (25)	2 (14.3)	0.635
**History of antibiotics usage within 2 months**	N (%)	12 (100)	12 (85.7)	0.483
Exposed days of antibiotics	Median (1Q, 3Q)	18 (8.5, 41.3)	8.5 (3.8, 23)	0.143*
Total days of antibiotics (sum of days for each antibiotics)	Median (1Q, 3Q)	22.5 (10.5, 66.3)	11 (3.8, 30.8)	0.089*
**Class of antibiotics**	N (%)			
Broad spectrum cephalosporin		6 (50)	9 (64.3)	0.462
Fluoroquinolones		5 (41.7)	1 (7.1)	0.065
Carbapenem		5 (41.7)	4 (28.6)	0.683
ß-lactam/ß-lactamamase inhibitor		5 (41.7)	6 (42.9)	0.951
Glycopeptides		2 (16.7)	3 (21.4)	1
**Clinical response**	N (%)	N = 11	N = 13	
Cure at the end of treatment		7 (63.6)	13 (100)	0.031
Failure		1 (9.1)	0	
Mortality		3 (27.3)	0	0.082
Attributable mortality		0	0	
Recurrence		2 (18.2)	5 (38.5)	0.386
Global cure		5 (45.5)	8 (61.5)	0.682

*P* value by Pearson’s chi-square test, *P value by Mann–Whitney U-test.

^1^WBC count > 15,000 cells/mm^3^.

^2^Albumin level < 2.5 mg/dL.

^3^Temperature > 38.3 °C.

^4^Elevated serum creatinine: > 1.5 baseline.

^5^Sum of 2 factors ≥ 1: leukocytosis and acute kidney injury.

^6^Sum of 4 factors ≥ 2: age over 60, leukocytosis, fever, and hypoalbuminemia.

## Discussion

In this study, we measured the relative abundance of *C. difficile* in the gut microbiome of CDI patients using metagenome sequences of gut microbiome. The gene *tcdB* was used as a target gene to estimate the relative abundance of toxigenic *C. difficile* in the gut microbiome. In general, quantitation of *C. difficile* culture presented as CFU (colony forming unit)/g faeces, or quantitative PCR of stool is used to evaluate the burden of *C. difficile*^9,10^. Our metagenomic approach applied in this study has an advantage over such existing methods in the sense that it can calculate the proportion of *C. difficile* population in the gut microbiome. Interestingly, the relative abundance of toxigenic *C. difficile* in the gut microbiome ranged from undetectable to 2.82% of the organisms in the gut, and in 12 of the 26 patients (46%) the *C. difficile* population amounted to > 0.1% of the gut microbiome. The mammalian gut is colonized by trillions of microorganisms^14^, and although the number of microorganisms in the gut might be reduced due to antibiotic use that predisposed the patients to CDI, the *C. difficile* population in the gut of patients with CDI might approach to billions.

Several studies have shown that the burden of *C. difficile* was not associated with the severity of CDI, and that the clinical severity of CDI was associated with the inflammatory response in the gut and the virulence of infecting organisms in human and mice^9,10,15^. The relative abundance of the organisms did not correlate with clinical severity in our study as well. However, unexpectedly, the *C. difficile* burden showed a negative correlation with the occurrence of fever and the treatment duration. This finding indicates that patients with sufficient immunity might have developed an inflammation to cause fever so as to control the pathogen levels in their gut, and the treatment duration was prolonged due to the inflammation in their gut.

We analysed the taxonomic composition of the intestinal microbiome using whole metagenome sequencing. The change in the bacterial composition of the CDI patients was similar to that reported in previous studies, which used amplification of 16S rRNA gene for identifying microbial taxa^3–5,16–18^. Among many genera that are known to be enhanced in healthy people compared with CDI patients, it is interesting that only the genera of *Bifidobacterium* and *Bacteroides* showed a significant negative association with toxigenic *C. difficile* when analysed using the proportion in the gut microbiome.

Age, gender, and underlying diseases are known to influence the microbiome structures of gut^11,14^, and hospitalization and usage of antibiotics have a huge impact^11^. In this study, the age and underlying diseases were not matched between the patients with CDI and healthy people. In addition, hospitalization and antibiotic usage were not matched because of the inclusion criteria of healthy people, which might contribute to the difference in the structure of gut microbiome and distribution of ARGs between CDI patients and healthy people.

We found that the bacterial composition is an important discriminator to cluster the patients of CDI into two groups: one group with high abundance of *Enterococcus,* and the other with high abundance of *Bacteroides* or *Lactobacillus*. Compared with the previous report based on 16S rRNA amplicon sequencing data^5^, bacterial composition patterns were generally consistent, but we further characterized the two groups clinically and microbiologically. The bacterial diversity was significantly lower in *Enterococcus* cluster, which suggests that gut microbiome structure was more destroyed. The total days of antibiotic usage were marginally higher in *Enterococcus* cluster. Since we counted the antibiotic usage only within 2 months from the enrolment, we suspect that probably more antibiotics might have been used in the cluster. Interestingly, more clinical cure was achieved in *Bacteroides* cluster, and all fatal cases came from the *Enterococcus* cluster despite no significant difference in demographics, comorbidities, and clinical severity of the diseases. These findings suggest a poor prognosis of *Enterococcus* cluster with more destruction of gut microbiome structure.

In terms of ARGs in gut microbiome, a four-fold increase in the number of ARGs was detected in CDI patients relative to that in healthy people. Recent admission history and antibiotic usage in CDI patients would contribute to the enrichment of ARGs in gut microbiome of CDI patients. Above all, class A β-lactamase genes, which include clinically important extended-spectrum β-lactamase genes, were markedly increased with the enhancement of *Enterobacteriaceae* in patients with CDI. Furthermore, plasmid-mediated carbapenemase genes were detected in three patients. A marked increase in ARGs along with high carriage number of *C. difficile* organisms in the gut of CDI patients reinforced the necessity of contact precaution of CDI patients.

To the best of our knowledge, this is the first attempt to investigate the association of the abundance of *C. difficile* with the clinical and microbiological characteristics in gut microbiome. The distribution of ARGs in the gut microbiome of CDI patients was compared with that of healthy individuals for the first time as well. Despite the methodological advantages, there are certain limitations in this study. The number of enrolled patients is relatively small, and healthy controls were not matched with CDI patients in age, underlying diseases, and antibiotic usage.

To summarize, the population of *C. difficile* in the gut of patients with CDI varied significantly, but did not influence the clinical severity. Regarding the bacterial composition in the gut, the patients of CDI could be discriminated into *Enterococcus*-rich clusters with low bacterial diversity, and *Bacteroides*-rich clusters with preserved bacterial diversity, and the patients belonging to the latter cluster led to a better clinical cure.

## Methods

### Study design and sample collection

The study was conducted at the Hanyang University Seoul Hospital, a 900-bed tertiary care facility and the Hanyang University Guri Hospital, a 600-bed secondary care facility, in South Korea. Through July 2016 to June 2018, individuals diagnosed with CDI were screened. CDI patients who could provide subsample over 50 g of faeces from CDI diagnosed stool sample were enrolled.

For the comparison, existing metagenomic data of 61 healthy Korean individuals were used^19^. Briefly, healthy person was defined as zero score according to the Charlson comorbidity index^20^ and no admission history within the past year, and individuals aged between 30 and 59 were enrolled from June to October 2017 at the Hanyang University Health Promotion Center for the health screening services.

The institutional review boards of Hanyang University Hospital and Hanyang University Guri Hospital approved these protocols, and written informed consent was obtained from all the participants. All methods were performed in accordance with relevant guidelines and regulations.

### Definitions and collection of data

CDI was diagnosed when the *C. difficile* isolates from stool culture showed positive toxin genes (*tcdA, tcdB, cdtA*, or *cdtB*) by multiplex PCR, positive results in toxin assay A&B with commercial kit (VIDAS C. difficile Toxin A & B; BioMerieux SA, Marcy l’Etoile, France), and/or pseudomembrane on endoscopy^21^. Real-time PCR for *tcdB* was performed with Xpert CD assay (Cepheid, USA) according to manufacturer’s instructions; cyclic threshold (Ct) value was used for semi-quantitative analysis of *tcdB* in the stool. Demographic and clinical characteristics data were collected retrospectively using medical records^20^. The severity of CDI was assessed by two methods as described^12,13^. Recurrence was defined as the resurgence of symptoms with diagnosis as CDI after cessation of treatment, at least 10 days after the first episode. Global cure was defined as patients who were cured at the end of treatment and did not have a recurrence^22,23^.

### Faecal DNA preparation, sequencing, and sequence filtering

Faeces was collected into a sterile container and stored at −80 °C deep freezer prior to DNA extraction. Methods for faecal DNA preparation, sequencing, and sequence filtering were followed by Human Metagenome Project- core microbiome sampling protocol A^24^. Total DNA was extracted using the Fast DNA SPIN Kit for Feces (MP Biomedicals, #116,570,200) and Illumina HiSeqX Platform (Illumina, San Diego, USA) was used to sequence the samples. Low-quality reads were removed using Sickle^25^. Four Gb of read sequences were retained in each sample for further quantitative analysis.

### Metagenomic analysis of tcdB, taxonomic composition and antibiotic resistance genes

Filtered reads were assembled into contigs using MEGAHIT^26^ with default options. Genes were predicted from contigs (> 500 bps) using FragGeneScan^27^. MetaPhlAn^28,29^, which uses clade-specific marker genes to profile bacterial compositions with the whole metagenome sequencing data, was used to find the taxonomic composition of each sample. The relative abundance of *C. difficile* was also reported based on this profile result. The Partitioning Around Medoids (PAM) algorithm in R package was used to cluster the samples based on bacterial composition. The optimal number of clusters was selected as two after calculating the *silhouette* score (= 0.36). The relative abundance of *tcdB* (accession no. NC_009089.1:786021-805508) was measured by mapping reads to the genes using Bowtie to measure toxigenic *C. difficile.* The relative abundance was reported by RPKM^30^. To identify ARGs, genes predicted in the metagenomic data set were searched against the ARGs annotated in the CARD database^31^ using Blastp^32^ with the threshold of an e-value less than 1 × 10^–10^, similarity over 70%, and reference coverage over 70%. The resistance genes were classified into 53 ARG subclasses and 20 ARG classes based on the gene ontology^31^ (Supplementary Table S4). For normalization, RPKM (read per kilobase million read) was used for measuring the abundance of *tcdB*; GPM (gene per million genes) was used as a measure of the abundance of ARGs in each sample:

$$ {\text{GPM}} = \frac{{{\text{Number}}\;{\text{of}}\;{\text{ARGs}}\;{\text{annotated}}}}{{{\text{Number}}\;{\text{of}}\;{\text{genes}}\;{\text{predicted}}}}*10^{6} . $$

The images for figures were generated by using R package.

### Statistical analysis

To compare the demographics and clinical characteristics, SPSS version 24.0 for Windows (SPSS Inc., Armonk, NY, USA) was used. Pearson’s chi-square test or Fisher’s exact test were used to analyse categorical variables, and Mann–Whitney U-test was used to analyse continuous variables. Spearman’s rank correlation test was performed to evaluate the relationship between two variables. A *p* value of < 0.05 by a two-tailed test was considered to be statistically significant.

### Ethics approval and consent to participate

The study protocol was approved by the institutional review boards (IRB No. HYUH 2016-05-031 and HYUH 2017-06-001 from Hanyang University Hospital and GURI 2016-05-003 from Hanyang University Guri hospital), and written informed consent was obtained from all the participants.

### Consent of publication

All participants in this study provided consent for publication.

## Supplementary information

Supplementary information1

Supplementary file2

## Data Availability

All raw sequencing data described in this study is available at European Nucleotide Archive (ENA) with the accession Nos. PRJEB35738 and PRJEB33013.
