# Supplementary material for: Quantitative characterization of Clostridioides difficile population in the gut microbiome of patients with C. difficile infection and their association with clinical factors
Source: Sci Rep. 2020 Oct 19;10:17608. doi: 10.1038/s41598-020-74090-0 (PMC7573688; doi:10.1038/s41598-020-74090-0)
Supplement: Supplementary file 2 — Supplementary file2 [file 41598_2020_74090_MOESM2_ESM.docx]

Title page

Title: Quantitative characterization of *Clostridioides difficile* population in the gut microbiome of patients with *C. difficile* infection and their association with clinical factors

Running title: Abundance of *C. difficile* in gut microbiome

Jieun Kim

Department of Internal Medicine, College of Medicine, Hanyang University, Seoul, 04763, Republic of Korea

Youna Cho

Department of Computer Science and Engineering, Hanyang University, Seoul, 04763, Republic of Korea

Mi-Ran Seo

Department of Internal Medicine, College of Medicine, Hanyang University, Seoul, 04763, Republic of Korea

Mi Hyun Bae

Department of Laboratory Medicine, College of Medicine, Hanyang University, Seoul, 04763, Republic of Korea

Bongyoung Kim

Department of Internal Medicine, College of Medicine, Hanyang University, Seoul, 04763, Republic of Korea

Mina Rho*

Department of Computer Science and Engineering, Hanyang University

Department of Biomedical Informatics, Hanyang University, Seoul, 04763, Republic of Korea^5^

Hyunjoo Pai*

Department of Internal Medicine, College of Medicine, Hanyang University, Seoul, 04763, Republic of Korea

Corresponding author

Hyunjoo Pai*

Tel: 82-2-2290-8356, Fax: 82-2-2298-9183

E-mail address: [paihyunjoo@gmail.com](about:blank)

Mina Rho*

Tel: 82-2-2220-2379

E-mail address: minarho@hanyang.ac.kr

*Equally contributed as the corresponding author

Supplementary Table S1. Genus-level bacterial composition of gut microbiome in the 26 patients with *Clostridioides difficile* infection compared with that in 61 healthy people

| Genus | Healthy person | CDI patients | *P* value |
| --- | --- | --- | --- |
|  | Median (1Q, 3Q) | Median (1Q, 3Q) |  |
| *Enterococcus* | 0 (0, 0.01822) | 24.08758 (6.67552, 59.30795) | <0.001 |
| *Bifidobacterium* | 12.4921 (7.00042, 22.33582) | 0.09919 (0.01584, 3.33747) | <0.001 |
| *Bacteroides* | 3.38671 (1.20546, 7.61807) | 1.19442 (0.01790, 8.36113) | 0.204 |
| *Ruminococcus* | 7.76156 (3.63953, 13.36869) | 0 (0, 0.00046) | <0.001 |
| *Eubacterium* | 7.34904 (3.67028, 10.55559) | 0 (0, 0.45389) | <0.001 |
| *Faecalibacterium* | 7.00874 (4.07817, 11.41992) | 0 (0, 0.02639) | <0.001 |
| *Lactobacillus* | 0.22884 (0.04189, 2.27408) | 2.46923 (0.10236, 10.8208) | 0.031 |
| *Prevotella* | 0.4976 (0.00469, 5.10012) | 0 (0, 0.15427) | 0.001 |
| *Blautia* | 3.24478 (2.04967, 5.16437) | 0.83352 (0, 3.95474) | 0.003 |
| *Subdoligranulum* | 2.0789 (1.35112, 4.54794) | 0.03084 (0.00281, 1.7116) | <0.001 |
| *Collinsella* | 2.73233 (1.44361, 5.80536) | 0 (0, 0) | <0.001 |
| *Escherichia* | 0.0789 (0.01696, 0.53300) | 1.95235 (0.15227, 4.61722) | 0.002 |
| *Akkermansia* | 0 (0, 0.14426) | 0.01248 (0, 0.05219) | 0.397 |
| *Megamonas* | 0.00769 (0, 1.24713) | 0 (0, 0) | <0.001 |
| *Dorea* | 2.94040 (1.57248, 5.23883) | 0 (0, 0.00285) | <0.001 |
| *Streptococcus* | 0.67756 (0.24146, 1.34826) | 0.59346 (0.02210, 2.68988) | 0.422 |
| *Alistipes* | 0.90998 (0.11811, 3.53922) | 0 (0, 0.14786) | <0.001 |
| *Erysipelotrichaceae_noname* | 1.0412 (0.02102, 3.30584) | 0.33155 (0.01562, 0.97059) | 0.13 |
| *Lachnospiraceae_noname* | 1.45377 (0.64952, 2.74908) | 0.04203 (0, 0.48693) | <0.001 |
| *Dialister* | 0 (0, 2.81176) | 0 (0, 0) | 0.003 |
| *Coprococcus* | 1.47349 (0.71307, 2.73530) | 0 (0, 0) | <0.001 |
| *Klebsiella* | 0 (0, 0.03565) | 0.65733 (0.04832, 3.56970) | <0.001 |
| *Catenibacterium* | 0.01187 (0, 2.72507) | 0 (0, 0) | <0.001 |
| *Parabacteroides* | 0.55950 (0.10386, 0.98594) | 0.03926 (0, 2.5799) | 0.142 |
| *Veillonella* | 0.00894 (0, 0.12014) | 0.05242 (0, 1.41023) | 0.077 |

*p*-value by Mann Whitney U-test

CDI; *Clostridioides difficile* infection, Median; median abundance (%) of each genus

Supplementary Table S2. Family-level bacterial composition of the gut microbiome in the 26 patients with *Clostridioides difficile* infection compared with that of 61 healthy people

| family | Healthy person | CDI patients | *P* value |
| --- | --- | --- | --- |
|  | Median (1Q, 3Q) | Median (1Q, 3Q) |  |
| *Ruminococcaceae* | 19.17089 (12.57137, 23.00854) | 0.48967 (0.01499, 3.30331) | <0.001 |
| *Enterococcaceae* | 0 (0, 0.01822) | 24.08758 (6.67552, 59.30795) | <0.001 |
| *Bifidobacteriaceae* | 12.4921 (7.00042, 22.33582) | 0.09919 (0.01584, 3.36273) | <0.001 |
| *Lachnospiraceae* | 14.06684 (8.62814, 18.09351) | 1.91089 (0.03665, 5.06226) | <0.001 |
| *Bacteroidaceae* | 3.38671 (1.20546, 7.61807) | 1.19442 (0.0179, 8.36113) | 0.204 |
| *Lactobacillaceae* | 0.25588 (0.05139, 2.27408) | 3.39527 (0.10236, 17.77915) | 0.012 |
| *Eubacteriaceae* | 7.34904 (3.69884, 10.55559) | 0 (0, 0.46524) | <0.001 |
| *Veillonellaceae* | 2.20068 (0.26968, 9.4683) | 0.07912 (0, 3.02639) | 0.002 |
| *Enterobacteriaceae* | 0.1435 (0.04776, 0.83697) | 4.47928 (2.18616, 16.91019) | <0.001 |
| *Prevotellaceae* | 0.95851 (0.07347, 5.6442) | 0.00126 (0, 0.15427) | <0.001 |
| *Coriobacteriaceae* | 3.33888 (2.31755, 6.86804) | 0.57649 (0.00955, 1.44746) | <0.001 |
| *Erysipelotrichaceae* | 3.14138 (0.12841, 6.49837) | 0.64921 (0.04862, 1.90341) | 0.014 |
| *Verrucomicrobiaceae* | 0 (0, 0.14426) | 0.01248 (0, 0.05219) | 0.397 |
| *Streptococcaceae* | 0.70903 (0.27153, 1.47727) | 0.59346 (0.02231, 2.69122) | 0.295 |
| *Rikenellaceae* | 0.90998 (0.11811, 3.53922) | 0 (0, 0.14786) | <0.001 |
| *Porphyromonadaceae* | 0.67975 (0.15728, 1.40109) | 0.0848 (0, 3.61409) | 0.136 |

*p*-value by Mann Whitney U-test

CDI; *Clostridioides difficile* infection, Median; median abundance (%) of each family

Supplementary Table S3. Microbial families and genera associated with the abundance of *tcdB* in the gut microbiome

| family | rho | *P* value | genus | rho | *P* value |
| --- | --- | --- | --- | --- | --- |
| *Bacteroidaceae* | -0.403 | 0.041 | *Alistipes* | -0.087 | 0.673 |
| *Bifidobacteriaceae* | -0.411 | 0.037 | *Bacteroides* | -0.403 | 0.041 |
| *Clostridiaceae* | -0.185 | 0.366 | *Bifidobacterium* | -0.417 | 0.034 |
| *Coriobacteriaceae* | -0.194 | 0.343 | *Blautia* | -0.093 | 0.65 |
| *Enterobacteriaceae* | 0.25 | 0.217 | *Collinsella* | -0.331 | 0.098 |
| *Enterococcaceae* | 0.163 | 0.428 | *Coprococcus* | -0.147 | 0.475 |
| *Erysipelotrichaceae* | -0.297 | 0.141 | *Dialister* | -0.026 | 0.899 |
| *Eubacteriaceae* | -0.145 | 0.478 | *Dorea* | 0.003 | 0.988 |
| *Lachnospiraceae* | -0.125 | 0.543 | *Enterococcus* | 0.163 | 0.428 |
| *Lactobacillaceae* | -0.209 | 0.306 | *Erysipelotrichaceae_noname* | -0.324 | 0.107 |
| *Porphyromonadaceae* | -0.136 | 0.508 | *Eubacterium* | -0.117 | 0.57 |
| *Prevotellaceae* | 0.153 | 0.456 | *Faecalibacterium* | -0.061 | 0.766 |
| *Rikenellaceae* | -0.087 | 0.673 | *Lachnospiraceae_noname* | -0.11 | 0.593 |
| *Ruminococcaceae* | -0.23 | 0.258 | *Lactobacillus* | -0.209 | 0.306 |
| *Streptococcaceae* | -0.071 | 0.731 | *Prevotella* | 0.205 | 0.315 |
| *Veillonellaceae* | 0.188 | 0.357 | *Ruminococcus* | -0.113 | 0.584 |
| *Verrucomicrobiaceae* | -0.051 | 0.804 | *Subdoligranulum* | -0.363 | 0.068 |

*p-*value by Spearman’s rho correlation analysis

Supplementary Table S4. Summary of determinants and the associated genes used in this study

| Determinant | Class | Gene Counts |
| --- | --- | --- |
| Aminocoumarin | antibiotic resistant DNA topoisomerase subunit | 1 |
| Aminoglycoside | aminoglycoside acetyltransferase (AAC) | 81 |
|  | aminoglycoside phosphotransferase (APH) | 34 |
|  | aminoglycoside nucleotidyltransferase (ANT) | 36 |
|  | 16S ribosomal RNA methyltransferase | 11 |
| Beta-lactam | class A beta-lactamase | 660 |
|  | class C beta-lactamase | 287 |
|  | class B (metallo-) beta-lactamase | 162 |
|  | class D beta-lactamase | 292 |
|  | general bacterial porin with reduced permeability to beta-lactams | 2 |
|  | beta-lactam resistant penicillin-binding proteins | 6 |
| Diaminopyrimidine | trimethoprim resistant dihydrofolate reductase dfr | 28 |
| Fluoroquinolone | quinolone resistance protein (qnr) | 95 |
| Fosfomycin | murA transferase | 1 |
|  | Fom phosphotransferase family | 2 |
|  | fosfomycin inactivation enzyme | 11 |
|  | fosC phosphotransferase family | 2 |
| Fusidic acid | fusidic acid inactivation enzyme | 4 |
| Glycopeptide | Bleomycin resistant protein | 1 |
|  | gene(s) or protein(s) associated with a glycopeptide resistance cluster | 51 |
| LMS | Cfr 23S ribosomal RNA methyltransferase | 6 |
|  | Erm 23S ribosomal RNA methyltransferase | 33 |
| Lincosamide | lincosamide nucleotidyltransferase (LNU) | 8 |
|  | LlmA 23S ribosomal RNA methyltransferase | 1 |
| Macrolide | macrolide inactivation enzyme | 16 |
|  | non-erm 23S ribosomal RNA methyltransferase (G748) | 4 |
|  | gimA family macrolide glycosyltransferase | 1 |
| Mupirocin | ileS | 3 |
| Nucleoside | streptothricin acetyltransferase (SAT) | 3 |
|  | tunicamycin resistance protein | 1 |
| Peptide | undecaprenyl pyrophosphate related proteins | 2 |
|  | defensin resistant mprF | 5 |
|  | Bah amidohydrolase | 1 |
|  | Edeine acetyltransferase | 1 |
|  | non-erm 23S ribosomal RNA methyltransferase (A1067) | 1 |
|  | viomycin phosphotransferase | 1 |
| Phenicol | chloramphenicol acetyltransferase (CAT) | 35 |
|  | chloramphenicol phosphotransferase | 1 |
| Polymyxin | phosphoethanolamine transferase conferring colistin resistance | 12 |
|  | lipid A phosphatase | 1 |
| Rifamycin | rifampin inactivation enzyme | 11 |
|  | rifamycin-resistant beta-subunit of RNA polymerase (rpoB) | 1 |
|  | rifampin-resistant RNA polymerase-binding protein | 1 |
| Streptogramin | streptogramin inactivation enzyme | 11 |
| Sulfonamide | sulfonamide resistant dihydropteroate synthase | 3 |
| Tetracycline | tetracycline-resistant ribosomal protection protein | 11 |
|  | tetracycline inactivation enzyme | 2 |
